# Comparisons Between Hypothesis- and Data-Driven Approaches for Multimorbidity Frailty Index: A Machine Learning Approach

**DOI:** 10.2196/16213

**Published:** 2020-06-11

**Authors:** Li-Ning Peng, Fei-Yuan Hsiao, Wei-Ju Lee, Shih-Tsung Huang, Liang-Kung Chen

**Affiliations:** 1 Aging and Health Research Center National Yang Ming University Taipei Taiwan; 2 Department of Geriatric Medicine National Yang Ming University School of Medicine Taipei Taiwan; 3 Center for Geriatrics and Gerontology Taipei Veterans General Hospital Taipei Taiwan; 4 Graduate Institute of Clinical Pharmacy College of Medicine National Taiwan University Taipei Taiwan; 5 School of Pharmacy College of Medicine National Taiwan University Taipei Taiwan; 6 Department of Pharmacy National Taiwan University Hospital Taipei Taiwan; 7 Department of Family Medicine Taipei Veterans General Hospital Yuanshan Branch Yi-Lan Taiwan

**Keywords:** multimorbidity frailty index, machine learning, random forest, unplanned hospitalizations, intensive care unit admissions, mortality

## Abstract

**Background:**

Using big data and the theory of cumulative deficits to develop the multimorbidity frailty index (mFI) has become a widely accepted approach in public health and health care services. However, constructing the mFI using the most critical determinants and stratifying different risk groups with dose-response relationships remain major challenges in clinical practice.

**Objective:**

This study aimed to develop the mFI by using machine learning methods that select variables based on the optimal fitness of the model. In addition, we aimed to further establish 4 entities of risk using a machine learning approach that would achieve the best distinction between groups and demonstrate the dose-response relationship.

**Methods:**

In this study, we used Taiwan’s National Health Insurance Research Database to develop a machine learning multimorbidity frailty index (ML-mFI) using the theory of cumulative diseases/deficits of an individual older person. Compared to the conventional mFI, in which the selection of diseases/deficits is based on expert opinion, we adopted the random forest method to select the most influential diseases/deficits that predict adverse outcomes for older people. To ensure that the survival curves showed a dose-response relationship with overlap during the follow-up, we developed the distance index and coverage index, which can be used at any time point to classify the ML-mFI of all subjects into the categories of fit, mild frailty, moderate frailty, and severe frailty. Survival analysis was conducted to evaluate the ability of the ML-mFI to predict adverse outcomes, such as unplanned hospitalizations, intensive care unit (ICU) admissions, and mortality.

**Results:**

The final ML-mFI model contained 38 diseases/deficits. Compared with conventional mFI, both indices had similar distribution patterns by age and sex; however, among people aged 65 to 69 years, the mean mFI and ML-mFI were 0.037 (SD 0.048) and 0.0070 (SD 0.0254), respectively. The difference may result from discrepancies in the diseases/deficits selected in the mFI and the ML-mFI. A total of 86,133 subjects aged 65 to 100 years were included in this study and were categorized into 4 groups according to the ML-mFI. Both the Kaplan-Meier survival curves and Cox models showed that the ML-mFI significantly predicted all outcomes of interest, including all-cause mortality, unplanned hospitalizations, and all-cause ICU admissions at 1, 5, and 8 years of follow-up (*P*<.01). In particular, a dose-response relationship was revealed between the 4 ML-mFI groups and adverse outcomes.

**Conclusions:**

The ML-mFI consists of 38 diseases/deficits that can successfully stratify risk groups associated with all-cause mortality, unplanned hospitalizations, and all-cause ICU admissions in older people, which indicates that precise, patient-centered medical care can be a reality in an aging society.

## Introduction

Population aging is a global phenomenon that poses various challenges to societies [1]. The health characteristics of older people and their health care service utilization differ greatly from those of younger adults [2], and frailty plays a pivotal role in the health of older people [3-5]. Frailty has been widely accepted as a geriatric syndrome that substantially increases the complexity of diseases and the burden of care [3-5]. In addition, frailty is recognized as an intermediate state between healthy and unhealthy states, and the potential reversibility of its nature highlights the importance of considering frailty when aiming to maintain the health of older people [6]. Moreover, frailty involves the coexistence of multiple comorbid conditions, such as polypharmacy, depression, cognitive impairment, falls, and malnutrition [7]. Therefore, the early identification of frailty and appropriate intervention remain the core of health care services for older people.

Despite the clinical significance of frailty, conceptual and operational definitions of frailty are inconsistent across studies [8]. Currently, the two most widely accepted approaches include the phenotypic approach for physical frailty and the frailty index based on the theory of cumulative deficits [9]. Although the definitions of frailty provided by the two approaches overlapped to some extent, the major discrepancy is in the prefrail group, such that physically prefrail subjects demonstrated a wide range on the frailty index. Nevertheless, both definitions remain the most widely accepted [10]. The theory of cumulative deficits proposed that aging may be characterized by the presence of cumulative deficits in various domains of health (eg, multimorbidity, functional assessment, and psychosocial perspectives) [9]. With a sufficient number of variables, the individual component of the frailty index was considered the same weight to constitute the frailty index. Researchers applied the theory of cumulative deficits to various data sets and validated the ability of the frailty index (FI) to predict adverse clinical outcomes [4,9]. Internationally, documented health care services data sets have been widely used to develop the FI for the prediction of health outcomes, and studies from different countries have all shown optimal results [5,11,12]. In the United Kingdom, researchers developed the electronic FI (eFI) using electronic medical records, which significantly predicted the mortality of older people [13,14]. Using similar principles, we developed the multimorbidity FI (mFI) using Taiwan’s National Health Insurance data set and significantly predicted mortality, hospitalizations, and admissions to critical care units [4]. However, it is always challenging to use data sets with large study samples and many variables to select appropriate variables to construct an FI and to optimally categorize the FI into risk classes. Both eFI and mFI adopted expert recommendations in the selection of variables, and the eFI and mFI were then categorized into quartiles for group comparisons, which is a widely accepted approach. Nonetheless, selecting variables based on expert recommendations may result in a failure to recognize previously unidentified associations. In addition, the quartile approach for risk group categorization may successfully be used to construct the prediction model, but the intergroup comparisons in survival analysis may overlap and fail to establish a clear distinction.

Therefore, this study aimed to develop the mFI by using machine learning methods that select variables based on the best fitness of the model. Furthermore, we aim to further establish 4 entities of risk using a machine learning approach and ensure the dose-response relationship and the best distinction between groups.

## Methods

### Study Design and Participants

This is a retrospective cohort study using data from Taiwan's National Health Insurance Research Database (NHIRD). Details about the NHIRD have been published [15]. Briefly, the NHIRD is a nationwide database composed of outpatient and inpatient claims, and it covers more than 99% of Taiwan's population. The data are checked for quality and maintained by the Data Science Centre of the Ministry of Health and Welfare of Taiwan. We used a subset of the NHIRD, which contains claims data for one million randomly selected beneficiaries from the Registry of Beneficiaries of the NHIRD in 2005. The study cohort consisted of 86,133 older adults aged 65 to 100 years who had full National Health Insurance (NHI) coverage from January 1, 2005, to December 31, 2005. Claims data from 2005 to 2013 for the one million beneficiaries was extracted to compose a 9-year (2005-2013) panel of claims for analysis. The study protocol was approved by the Research Ethics Committee of the National Taiwan University Hospital (NTUH-REC-201403069W).

### Construction of the Machine Learning–Based Multimorbidity Frailty Index

The mFI was constructed following standard procedures [16], and this method has been validated in the Taiwanese population [4,5]. Disease diagnoses (International Classification of Diseases, Ninth Revision, Clinical Modification [ICD-9-CM]) from outpatient and inpatient claims of the NHIRD between January 1 and December 31, 2005, were used to identify accumulated deficits to construct mFI. We adopted an algorithm widely used in studies using NHIRD as the data source to validate the diagnostic codes of the specified deficits within NHIRD; that is, only those who had at least 3 outpatient claim records or 1 inpatient claim record for that specified diagnosis code were considered to have the specified deficit. For example, an older adult must have at least 3 outpatient claim records or 1 inpatient claim record of diabetes mellitus [ICD-9-CM: 250] to be defined as having a deficit based on our definition.

**Figure 1:**
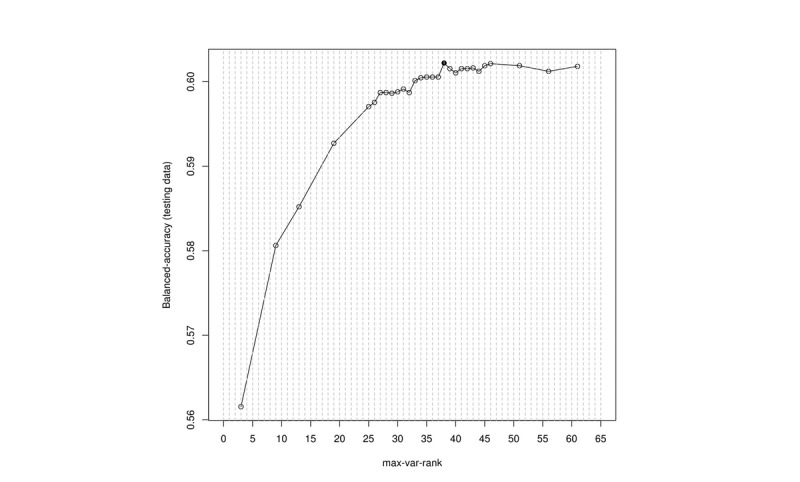
Numbers of diseases versus random forest model accuracy to determine an adequate number of frailty indexes.

A random forest method, with significant improvements in classification accuracy that resulted from growing an ensemble of trees and letting them vote for the most popular class, was adopted [17]. The variable importance of the random forest uses mean decrease accuracy to determine the specific conditions of machine learning–based multimorbidity frailty index (ML-mFI). The adequate constructive number of ML-mFI was 38 conditions, when the model accuracy reached the highest level, 0.602 (Figure 1 and Multimedia Appendix 1). The ML-mFI was calculated as the number of conditions a person encountered in a year out of the 38 selected ones.

### Determination of Frailty Status by ML-mFI

All subjects were further categorized into 4 entities (fit, mild frailty, moderate frailty, and severe frailty) based on their risk status; this categorization was used by a previous study [4]. The fundamental rules for risk stratification included the following: (1) the individual risk groups were significantly different from each other, and (2) the health risk of these groups showed a dose-response relationship (ie, those in the severe frailty group had a higher risk than those in the moderate frailty group, who had a higher risk than those in the mild frailty group, and so on, at any follow-up time point after the first year). To achieve this purpose, we developed two indices, the distance index and the coverage index, which ensured the distinction and dose-response relationship of all survival curves.

The distance index measured the distance between each survival curve and the stability of those distances within groups. At any time point, the distance index was defined as 
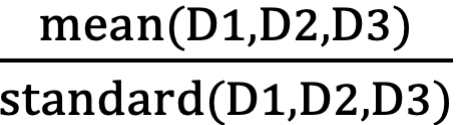
. Therefore, the distances within groups are wider and more stable when the distance index is larger (Multimedia Appendix 2). Conversely, the coverage index aimed to evaluate the length of the confidence interval for each survival curve. The total length of the confidence intervals indicated the overall estimated error in the grouping method. In Multimedia Appendix 3, the coverage index was defined as 
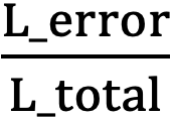
 at any individual time point, where L_total measured the difference in the estimated survival probability between the fit group and the severe frailty group, and L_error measured the total estimated errors within the 4 groups. When the coverage index is smaller, the estimated error within groups is smaller. With the application of both the distance index and the coverage index, the levels of frailty were successfully categorized into 4 groups by values of ML-mFI: fit was indicated by 0≤ML-mFI<0.026; mild frailty was 0.026≤ML-mFI<0.105; moderate frailty was 0.105≤ML-mFI<0.157; and severe frailty was 0.157≤ML-mFI. In the survival analysis, the grouping strategy successfully categorized all subjects into 4 groups with significant distinction during the follow-up period. In other words, there were no overlaps between the survival curves and the dose-response relationship between groups was clearly shown.

### Outcomes of Interest

The outcomes of interest in this study include all-cause mortality, unplanned hospitalizations, and intensive care unit (ICU) admissions. The date of mortality was identified as the date of disenrollment from the NHIRD, which has been validated in a previous study [4]. Unplanned hospitalizations were any unexpected hospitalizations after an emergency department visit. ICU admissions were any hospital admissions with the use of ICU services. All study subjects were continuously followed from January 1, 2006, to the occurrence of each outcome or the end of 2013, whichever came first. For the outcomes of unplanned hospitalizations and ICU admissions, subjects were censored at death if it occurred first. Preplanned analyses were conducted to evaluate the effectiveness of ML-mFI in predicting outcomes at 1, 5, and 8 years.

### Statistical Analysis

Numerical variables were expressed as the mean (SD), and categorical variables were expressed as a number or percentage. A random forest method not only determined the number of disease items comprising ML-mFI but also identified potential conditions of ML-mFI with prediction accuracy and variable importance. The distance index and coverage index with min-max and max-min criteria were used to determine cut points and categorize the frailty group by ML-mFI automatically. The Kaplan-Meier survival curve with the log-rank test was used to examine the association between categories of ML-mFI (fit, mild frailty, moderate frailty, and severe frailty) and 8-year mortality and hospitalizations. Cox proportional hazard models were used to estimate the hazard ratios (HRs) and 95% CIs for mortality and hospitalizations at 1, 5, and 8 years after the ML-mFI and mFI were estimated (based on a previous study [4]), considering both to be the independent variable. We further included age and gender as covariates in all adjusted models. Sex-specific analysis was conducted.

All of the analyses were performed using R Version 3.4.4 (R Foundation for Statistical Computing). A two-sided *P* value of <.05 was considered statistically significant. The coxph function in the survival package showed nonviolation of the proportional hazards assumption and a linear relationship between the log hazard and each covariate. The random forest and importance functions in the randomForest package showed the model building and variable importance to predict the outcome occurrence and comprise ML-mFI, respectively.

## Results

### Construction of ML-mFI

The final ML-mFI with the highest model accuracy (0.6022061) contained 38 conditions (Multimedia Appendix 1). Details of convergences and divergences of composing conditions among ML-mFI and mFI are shown in Multimedia Appendix 4. Table 1 compares the ML-mFI group and traditional mFI by age and sex. There were two similar distribution patterns on mFI and ML-mFI. ML-mFI increased with age, but reached a plateau at age 80 years and older. Both indices were higher in males, which is compatible with the shorter life expectancy of men in Taiwan. However, the mFI was calculated based on 32 selected conditions a person may have in a year, while the ML-mFI was calculated based on 38 selected conditions a person may have in a year; thus, the actual numbers on the mFI and ML-mFI were very different. Among people aged 65 to 69 years, the mean mFI and ML-mFI were 0.037 (SD 0.048) and 0.0070 (SD 0.0254), respectively. The difference may result from discrepancies in the conditions selected on the mFI and the ML-mFI. For example, some conditions were selected only on the ML-mFI but not on the mFI (eg, ICD-9-CM: 250 [diabetes mellitus] and, vice versa, ICD-9-CM: 374 [entropion]). These discrepancies have been shown in Multimedia Appendix 1.

**Table 1:** Comparisons of mFI and ML-mFI by age and sex.^a,b^

Age (years)	All subjects (N=86,133)		Male (n=42,914)		Female (n=43,219)	
	mFI, mean (SD)	ML-mFI, mean (SD)	mFI, mean (SD)	ML-mFI, mean (SD)	mFI, mean (SD)	ML-mFI, mean (SD)
65-69 (n=28,480)	0.037 (0.048)	0.0070 (0.0254)	0.038 (0.049)	0.0076 (0.0264)	0.037 (0.046)	0.0065 (0.0246)
70-74 (n=23,700)	0.050 (0.056)	0.0106 (0.0322)	0.053 (0.060)	0.0115 (0.0339)	0.046 (0.053)	0.0096 (0.0304)
75-79 (n=18,765)	0.062 (0.065)	0.0150 (0.0400)	0.067 (0.070)	0.0160 (0.0417)	0.056 (0.059)	0.0138 (0.0379)
80-84 (n=9934)	0.070 (0.071)	0.0201 (0.0473)	0.076 (0.075)	0.0212 (0.0490)	0.064 (0.065)	0.0190 (0.0455)
≥85 (n=5254)	0.070 (0.074)	0.0234 (0.0505)	0.077 (0.080)	0.0245 (0.0531)	0.064 (0.069)	0.0224 (0.0483)
Total (N=86,133)	0.052 (0.060)	0.0122 (0.0359)	0.056 (0.064)	0.0132 (0.0376)	0.048 (0.056)	0.0113 (0.0341)

^a^mFI: multimorbidity frailty index.

^b^ML-mFI: machine learning multimorbidity frailty index.

### Survival Analysis

**Figure 2:**
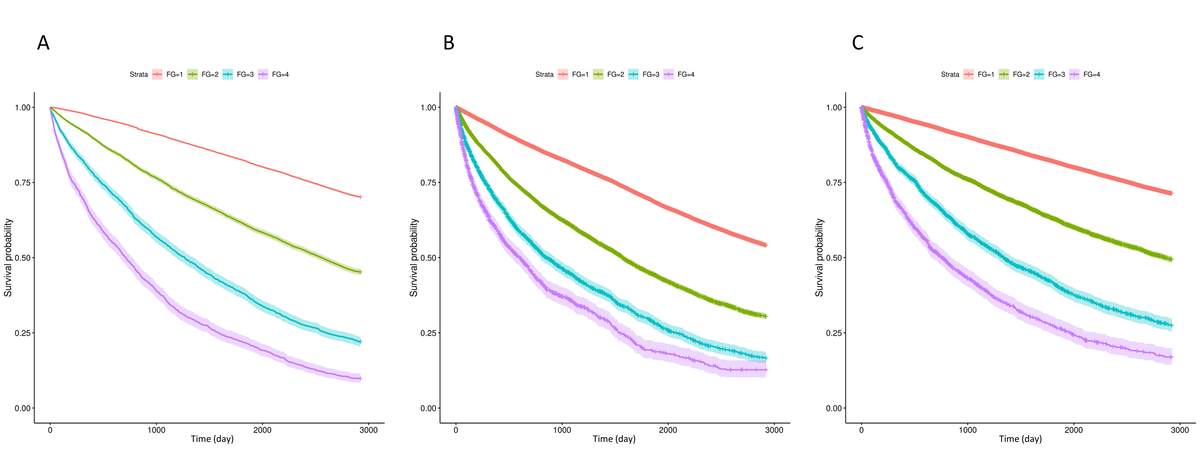
The 8-year Kaplan-Meier survival curve for the outcome of (A) all-cause mortality, (B) unplanned hospitalizations, and (C) intensive care unit admissions for different frailty categories.

Overall, 86,133 subjects aged 65 to 100 years were included in this study. With a mean follow-up of 6.57 (SD 2.37) years, 30,136 deaths (34.99%) occurred among the study cohort during the study period. Figure 2 summarizes the results of the Kaplan-Meier survival curves estimating 4 levels of ML-mFI on all-cause mortality, unplanned hospitalization, and ICU admission, and shows that ML-mFI significantly predicted all these outcomes of interest.

Table 2 shows the hazard ratios of all-cause mortality, unplanned admissions, and ICU admissions for the ML-mFI and the mFI at the 1-, 5- and 8-year follow-up periods. Among all three outcomes of interest, ML-mFI posed higher hazards than did mFI. For example, those who were categorized as severely frail by the mFI or the ML-mFI were associated with 4.97-fold (adjusted HR 4.97, 95% CI 4.49-5.50) and 11.4-fold (adjusted HR 11.40, 95% CI 10.32-12.59) increases in 1-year all-cause mortality, respectively. Similar patterns were observed for 5-year and 8-year all-cause mortality.

**Table 2:** Hazard ratios of all-cause mortality, unplanned hospitalizations, and intensive care unit admissions for the ML-mFI and the mFI at the 1-, 5- and 8-year follow-up periods.^a,b,c^ All values are given as hazard ratio (95% CI).

Adverse outcomes at follow-up periods		Mild frailty		Moderate frailty		Severe frailty	
		mFI (n=14,244)	ML-mFI (n=9366)	mFI (n=4741)	ML-mFI (n=2522)	mFI (n=2498)	ML-mFI (n=1488)
**1-year all-cause mortality HR^d^**							
	Unadjusted	2.21 (2.04-2.39)	3.66 (3.38-3.97)	4.09 (3.72-4.50)	8.81 (8.00-9.71)	7.52 (6.81-8.30)	16.62 (15.08-18.32)
	Adjusted	1.86 (1.71-2.01)	3.13 (2.89-3.39)	3.08 (2.80-3.39)	6.79 (6.15-7.49)	4.97 (4.49-5.50)	11.40 (10.32-12.59)
**5-year all-cause mortality HR**							
	Unadjusted	1.76 (1.70-1.82)	2.57 (2.48-2.67)	2.85 (2.72-2.99)	5.27 (5.00-5.55)	5.00 (4.74-5.28)	9.02 (8.49-9.58)
	Adjusted	1.46 (1.41-1.52)	2.19 (2.11-2.27)	2.14 (2.04-2.25)	4.04 (3.83-4.26)	3.28 (3.11-3.46)	6.15 (5.79-6.54)
**8-year all-cause mortality HR**							
	Unadjusted	1.69 (1.64-1.74)	2.32 (2.25-2.39)	2.65 (2.55-2.76)	4.72 (4.54-4.94)	4.50 (4.29-4.71)	8.05 (7.61-8.51)
	Adjusted	1.41 (1.37-1.45)	1.99 (1.93-2.05)	2.01 (1.93-2.09)	3.70 (3.53-3.88)	2.98 (2.84-3.12)	5.52 (5.22-5.84)
**1-year unplanned hospitalization HR**							
	Unadjusted	2.08 (1.97-2.20)	2.86 (2.70-3.02)	3.30 (3.07-3.54)	5.21 (4.82-5.64)	5.29 (4.88-5.73)	7.65 (6.99-8.38)
	Adjusted	1.91 (1.80-2.01)	2.63 (2.49-2.79)	2.85 (2.65-3.06)	4.53 (4.18-4.90)	4.28 (3.94-4.64)	6.20 (5.66-6.80)
**5-year unplanned hospitalization HR**							
	Unadjusted	1.78 (1.73-1.83)	2.28 (2.21-2.36)	2.51 (2.40-2.62)	3.79 (3.59-4.00)	3.85 (3.65-4.06)	5.43 (5.07-5.83)
	Adjusted	1.61 (1.57-1.66)	2.09 (2.02-2.16)	2.14 (2.05-2.24)	3.23 (3.06-3.41)	3.05 (2.89-3.23)	4.33 (4.04-4.65)
**8-year unplanned hospitalization HR**							
	Unadjusted	1.67 (1.63-1.71)	2.11 (2.05-2.17)	2.32 (2.24-2.41)	3.53 (3.36-3.71)	3.53 (3.36-3.71)	5.03 (4.69-5.38)
	Adjusted	1.51 (1.48-1.55)	1.93 (1.87-1.99)	1.98 (1.91-2.06)	3.01 (2.86-3.17)	2.79 (2.65-2.94)	3.98 (3.72-4.27)
**1-year intensive care unit admission HR**							
	Unadjusted	2.34 (2.18-2.52)	3.23 (3.00-3.48)	4.32 (3.95-4.72)	6.70 (6.08-7.38)	7.04 (6.38-7.76)	12.16 (10.98-13.46)
	Adjusted	2.09 (1.94-2.25)	2.91 (2.70-3.13)	3.59 (3.28-3.92)	5.64 (5.11-6.23)	5.35 (4.84-5.91)	9.41 (8.49-10.44)
**5-year intensive care unit admission HR**							
	Unadjusted	1.86 (1.79-1.93)	2.39 (2.30-2.48)	2.92 (2.78-3.07)	4.75 (4.48-5.04)	4.84 (4.56-5.14)	7.84 (7.30-8.42)
	Adjusted	1.64 (1.58-1.70)	2.14 (2.06-2.23)	2.42 (2.30-2.54)	3.96 (3.74-4.21)	3.65 (3.43-3.87)	6.00 (5.58-6.44)
**8-year intensive care unit admission HR**							
	Unadjusted	1.74 (1.69-1.79)	2.20 (2.12-2.28)	2.69 (2.58-2.81)	4.35 (4.12-4.59)	4.28 (4.05-4.52)	7.10 (6.63-7.60)
	Adjusted	1.54 (1.49-1.59)	1.98 (1.92-2.05)	2.23 (2.14-2.34)	3.68 (3.49-3.89)	3.24 (3.06-3.42)	5.46 (5.10-5.85)

^a^For all outcomes, the comparator is the study subjects in the fit categories (n=64,650). All data were adjusted for age and gender.

^b^ML-mFI: machine learning multimorbidity frailty index.

^c^mFI: multimorbidity frailty index.

^d^HR: hazard ratio.

For unplanned hospitalizations, those who were categorized as severely frail by the mFI or the ML-mFI were associated with 4.28-fold (adjusted HR 4.28, 95% CI 3.94-4.64) and 6.20-fold (adjusted HR 6.20, 95% CI 5.66-6.80) increases in 1-year unplanned hospitalizations, respectively. Similar patterns were observed for 5-year and 8-year all-cause unplanned hospitalizations.

For ICU admissions, those who were categorized as severely frail by the mFI or the ML-mFI were associated with 4.28-fold (adjusted HR 5.35, 95% CI 4.84-5.91) and 9.41-fold (adjusted HR 9.41, 95% CI 8.49-10.44) increases in 1-year ICU admissions, respectively. Similar patterns were observed for 5-year and 8-year all-cause ICU admissions.

Sex-specific analysis showed that both indices were higher in men than in women for various outcomes and follow-up periods (Tables 3 and 4 for males and females, respectively). For example, men in the severe frailty group (as defined by the ML-mFI) were associated with a 12.64-fold increased risk of 1-year mortality, while women in the severe frailty group (as defined by the mFI) were associated with a 10.37-fold increased risk of 1-year mortality.

**Table 3:** Male hazard ratios of all-cause mortality, unplanned hospitalization, and intensive care unit admission for ML-mFI and mFI among 1-, 5-, and 8-year follow-up periods.^a,b,c^

Adverse outcome and follow-up period		Mild frailty		Moderate frailty		Severe frailty	
		mFI (n=14,244)	ML-mFI (n=9366)	mFI (n=4,741)	ML-mFI (n=2522)	mFI (n=2498)	ML-mFI (n=1488)
**All-cause mortality**							
	1-year	1.83 (1.65-2.04)	3.71 (3.37-4.09)	2.70 (2.37-3.07)	7.67 (6.76-8.69)	4.84 (4.26-5.49)	12.64 (11.20-14.27)
	5-year	1.41 (1.35-1.48)	2.53 (2.42-2.65)	1.93 (1.82-2.06)	4.60 (4.29-4.93)	3.07 (2.86-3.28)	6.92 (6.40-7.48)
	8-year	1.35 (1.30-1.41)	2.29 (2.21-2.38)	1.85 (1.75-1.95)	4.19 (3.94-4.47)	2.77 (2.61-2.94)	6.27 (5.83-6.74)
**Unplanned hospitalization**							
	1-year	1.87 (1.73-2.01)	2.83 (2.64-3.04)	2.73 (2.48-3.00)	4.90 (4.41-5.45)	4.24 (3.83-4.71)	6.34 (5.63-7.14)
	5-year	1.58 (1.51-1.64)	2.25 (2.16-2.35)	2.05 (1.93-2.17)	3.49 (3.25-3.76)	3.00 (2.80-3.21)	4.59 (4.19-5.03)
	8-year	1.48 (1.43-1.53)	2.07 (1.99-2.15)	1.91 (1.81-2.01)	3.30 (3.08-3.54)	2.76 (2.59-2.95)	4.29 (3.92-4.69)
**Intensive care unit admission**							
	1-year	2.02 (1.83-2.23)	3.35 (3.06-3.67)	3.28 (2.91-3.69)	5.91 (5.18-6.74)	4.85 (4.27-5.51)	9.50 (8.31-10.86)
	5-year	1.58 (1.50-1.66)	2.42 (2.31-2.55)	2.24 (2.10-2.40)	4.33 (4.00-4.69)	3.39 (3.15-3.66)	6.28 (5.72-6.89)
	8-year	1.48 (1.42-1.54)	2.22 (2.13-2.32)	2.05 (1.94-2.18)	4.04 (3.75-4.35)	2.99 (2.79-3.21)	5.80 (5.30-6.35)

^a^For all outcomes, the comparator is subjects in fit categories (n=64,650). All data were adjusted for age and gender.

^b^ML-mFI: machine learning multimorbidity frailty index.

^c^mFI: multimorbidity frailty index.

**Table 4:** Female hazard ratios of all-cause mortality, unplanned hospitalization, and intensive care unit admission for ML-mFI and mFI among 1-, 5-, and 8-year follow-up periods.^a,b,c^

Adverse outcome and follow-up period		Mild frailty		Moderate frailty		Severe frailty		
		mFI (n=14,244)	ML-mFI (n=9366)	mFI (n=4741)	ML-mFI (n=2522)		mFI (n=2498)	ML-mFI (n=1488)
**All-cause mortality**								
	1-year	1.88 (1.66-2.13)	2.56 (2.28-2.87)	3.73 (3.22-4.32)	5.95 (5.19-6.82)		5.29 (4.46-6.27)	10.37 (8.97-12.00)
	5-year	1.54 (1.46-1.62)	1.87 (1.77-1.97)	2.52 (2.34-2.71)	3.51 (3.25-3.78)		3.79 (3.46-4.15)	5.52 (5.04-6.05)
	8-year	1.48 (1.42-1.55)	1.72 (1.65-1.80)	2.31 (2.17-2.46)	3.24 (3.04-3.46)		3.48 (3.22-3.76)	4.89 (4.49-5.32)
**Unplanned hospitalization**								
	1-year	1.95 (1.80-2.11)	2.45 (2.26-2.65)	3.03 (2.72-3.38)	4.17 (3.72-4.66)		4.36 (3.81-4.98)	6.19 (5.40-7.09)
	5-year	1.66 (1.59-1.73)	1.94 (1.86-2.03)	2.28 (2.14-2.44)	2.98 (2.77-3.21)		3.16 (2.89-3.46)	4.12 (3.71-4.58)
	8-year	1.56 (1.50-1.62)	1.81 (1.74-1.88)	2.10 (1.98-2.22)	2.74 (2.55-2.94)		2.85 (2.62-3.11)	3.71 (3.34-4.12)
**Intensive care unit admission**								
	1-year	2.18 (1.95-2.44)	2.48 (2.22-2.76)	4.09 (3.56-4.70)	5.39 (4.71-6.16)		6.44 (5.49-7.55)	9.73 (8.40-11.27)
	5-year	1.73 (1.64-1.82)	1.88 (1.77-1.99)	2.70 (2.50-2.91)	3.62 (3.33-3.93)		4.21 (3.82-4.65)	5.89 (5.29-6.56)
	8-year	1.62 (1.55-1.70)	1.77 (1.69-1.86)	2.54 (2.37-2.71)	3.34 (3.10-3.61)		3.79 (3.47-4.15)	5.27 (4.76-5.85)

^a^For all outcomes, the comparator is subjects in fit categories (n=64,650). All data were adjusted for age and gender.

^b^ML-mFI: machine learning multimorbidity frailty index.

^c^mFI: multimorbidity frailty index.

## Discussion

In this study, we successfully used a machine learning approach to define ML-mFI. Specifically, we selected disease/deficit items by the random forest method and ranked the importance of each individual disease accordingly. The selection of these diseases/deficits items to construct ML-mFI was driven solely by data, while the conventional mFI included disease/deficit items based on expert recommendations. Moreover, the combined use of the distance index and coverage index successfully distinguished 4 groups with dose-response risks of adverse outcomes. In epidemiological studies, researchers have often encountered similar challenges in selecting appropriate variables for analysis and optimally categorizing continuous variables into categorical variables for further comparisons. Traditionally, researchers need to search for literature support or adopt a generic approach to develop an optimal statistical model for data interpretation [18-20]. The hypothesis-driven approach for a research question is of great importance in scientific development; however, previously unknown or unidentified factors may be overlooked in the analysis, which may lower the statistical power in the interpretation of the phenomenon. Compared to our previous work where we used a hypothesis-driven approach to construct the mFI [4], the machine learning model selected significantly different disease/deficit items for ML-mFI construction. The traditional approach selected the diseases/deficits of older adults based on the selection criteria, and the machine learning approach identified more disease/deficit items, including chronic diseases, infectious diseases, and even some cancers, but these items did not comprise the majority of disease/deficit items.

The FI developed by Rockwood et al [18] hypothesized that cumulative deficits in various health domains may represent the process of biological aging, and this FI has been widely validated to predict adverse health events and mortality in different countries [5,11,12]. In theory, an FI may consist of as many variables as possible, so there are no issues regarding variable selection. However, to meet the needs of the busy clinical environment, the mFI is derived from the concept of the FI; a selection of age-related chronic conditions were the key variables used to construct the prediction model. Existing studies have shown that these previously developed mFIs can significantly predict the mortality of older adults [13.14]. However, to maximize the effectiveness of the prediction model, using a data-driven approach to construct the ML-mFI may provide better prediction accuracy. Moreover, in the survival analysis, the dose-response relationship is usually expected when grouping continuous measurements into distinct risk groups in association with outcomes. However, the distinction between individual groups from the continuous measurements is not always statistically significant even though the whole model reached statistical significance. For example, in one Taiwanese study, the developed FI was found to predict the adverse outcomes of older adults, which was in line with most related studies [5]. However, in that study, different risk groups that were categorized based on FI tertiles resulted in overlapping survival curves of the intermediate- and high-risk groups; it failed to achieve the stratification of risk groups. The combined use of the distance index and coverage index developed in this study engenders the ability to address the overlapping phenomenon of survival curves.

Although the mFI we developed adequately predicted adverse outcomes for older adults, the ML-mFI showed relatively higher hazard ratios than did the mFI for all health outcomes. Overall, the data-driven ML-mFI may identify different at-risk populations than the hypothesis-driven mFI. The data-driven approach may disclose the phenomenon of the whole data set [21-23], but the hypothesis-driven approach may provide a better explanation for the observations [24,25]. The data-driven approach may not be superior to the hypothesis-driven approach, since the study purpose and research questions may vary greatly. Although a data-driven approach may usually establish a prediction model with better accuracy, it is difficult to implement intervention programs for the observed phenomenon. Applying the theory of cumulative deficits, a large number of variables may be used to construct the prediction model, but it becomes challenging to further utilize the prediction model with a large number of variables. Therefore, researchers have attempted to reduce the number of variables while maintaining optimal prediction accuracy. Our previous study used factor analysis to reduce the 125 selected variables into 35 factors to improve the clinical application [5]. However, the machine learning approach in this study may play a similar role in reducing the selected number of variables and optimizing prediction accuracy. The main strength of this study was to demonstrate the methodological advance of processing a large data set to select appropriate variables to construct a prediction model and to ensure the distinction of different risk groups with dose-response relationships. This methodological advance may facilitate public health or social sciences research, or interdisciplinary research that uses a large data set with a wide array of data characteristics. In particular, the distance index and coverage index would be of great importance for future research to categorize the results of continuous variables into distinct entities with different health risks. Avoiding the overlap of the survival curves of different risk groups by using the distance index and coverage index is important to strengthen the observed phenomenon and the risk group classification.

Therefore, this ML-mFI demonstrated an automatic approach to predict adverse outcomes in older people, and it can be applied to different populations in different countries. Using the same approach, different diseases can be selected to construct the new ML-mFI in another population to predict adverse outcomes in the corresponding population. For example, we further stratified our study population into 3 subcohorts, including those aged 65 to 75 years, 76 to 85 years, and 85 years and older, and we constructed three kinds of ML-mFI for each age group according to the same automatic machine learning approach and model selection criteria. We found that the total deficit number and composing deficits on the ML-mFI, as well the cut-off points of different frailty statuses, are quite different in distinct age groups. For example, the total deficit numbers on the ML-mFI were 59, 47, and 39 for those who were aged 65 to 75 years, 76 to 85 years, and 85 years and older, respectively. In addition, the composing deficits were different, as displayed in Multimedia Appendix 5. Although the composing deficits of the ML-mFI are different in distinct age groups, all of these ML-mFI can successfully predict all-cause mortality (C index>0.6). These findings are inspiring because they indicate that the same machine learning approach can be used to construct one’s own ML-mFI to fulfill this purpose. Individual diseases may have different clinical impacts in different countries due to diagnosis, treatment, and quality of care. Therefore, the results of this study can be applied to different countries and populations using the same approach to construct their own ML-mFI to meet their needs.

Therefore, our ML-mFI could have clinical implications in public health or in health care administration. For example, in large long-term care facilities management, the administration needs to optimize the admission waiting list through the estimation of the mortality of all residents. On the other hand, in public health settings, the government is able to accurately estimate the health risk of residents in a certain geographic area and to provide optimal health care or palliative care services. Traditionally, these decisions were made based on existing medical knowledge, but a data-driven approach may better predict outcomes and optimize the government’s public health policy. In clinical practice, the ML-mFI may enable physicians and families to quantify health risks for optimal care planning. Hence, using available electronic medical records, the ML-mFI can be automatically generated and integrated as part of the medical record to facilitate certain forms of decision-making in care planning.

Despite all the effort that went into this study, there are still some limitations. First, like all data-driven studies, the results of this study could not provide or validate a well-established hypothetical framework due to the nature of machine learning. Second, it remained difficult to develop further intervention programs based on the diagnostic entities identified by machine learning. Third, another data set is needed to examine whether overfitting exists in the machine learning model. Finally, as in most of the previous frailty index studies, although we adjusted for age and sex as covariates in the Cox model, we were unable to access some residual confounders not routinely captured in a claims database, such as disease severity or lifestyle factors (eg, physical activity and diet).

In conclusion, the ML-mFI significantly predicted adverse health outcomes for older adults, and the risk groups defined by the combination of the distance index and coverage index distinguished the different risk groups with dose-response relationships and clear distinctions. The methodological advance of this study also had further research implications for studies with similar data and research questions. The data-driven approach may provide better prediction accuracy than the hypothesis-driven approach, but the superiority of the data-driven approach requires further study for confirmation.

## Appendix

Multimedia Appendix 1 Model accuracy with various numbers of composite diseases.

Multimedia Appendix 2 Survival curves with 4 frail groups to depict the calculation of the distance index.

Multimedia Appendix 3 Survival curves with 4 frail groups to depict the calculation of the coverage index.

Multimedia Appendix 4 Convergences and divergences between mFI and ML-mFI.

Multimedia Appendix 5 Total number of deficits and the composing deficits of the ML-mFI in three subcohorts, including 65 to 75 years old, 76 to 85 years old, and >85 years old.

## Abbreviations

eFI: electronic frailty index
FI: frailty index
ICD-9-CM: International Classification of Diseases, Ninth Revision, Clinical Modification
ICU: intensive care unit
HR: hazard ratio
mFI: multimorbidity frailty index
ML-mFI: machine learning multimorbidity frailty index
NHI: National Health Insurance
NHIRD: National Health Insurance Research Database

## References

[1] Chen LK, Inoue H, Won CW, Lin CH, Lin KF, Tsay SF, Lin PF, Li SH (2013). Challenges of urban aging in Taiwan: Summary of urban aging forum. Journal of Clinical Gerontology and Geriatrics.

[2] Lu WH, Lee WJ, Chen LK, Hsiao FY (2016). Comparisons of annual health care utilization, drug consumption, and medical expenditure between the elderly and general population in Taiwan. Journal of Clinical Gerontology and Geriatrics.

[3] Lee WJ, Peng LN, Lin CH, Lin HP, Loh CH, Chen LK (2018). The synergic effects of frailty on disability associated with urbanization, multimorbidity, and mental health: implications for public health and medical care. Sci Rep.

[4] Wen YC, Chen LK, Hsiao FY (2017). Predicting mortality and hospitalization of older adults by the multimorbidity frailty index. PLoS One.

[5] Lin SY, Lee WJ, Chou MY, Peng LN, Chiou SY, Chen LK (2016). Frailty Index Predicts All-Cause Mortality for Middle-Aged and Older Taiwanese: Implications for Active-Aging Programs. PLoS One.

[6] Van der Elst M, Schoenmakers B, Duppen D, Lambotte D, Fret B, Vaes B, De Lepeleire J, D-SCOPE Consortium (2018). Interventions for frail community-dwelling older adults have no significant effect on adverse outcomes: a systematic review and meta-analysis. BMC Geriatr.

[7] Chen LK, Hwang AC, Liu LK, Lee WJ, Peng LN (2016). Frailty Is a Geriatric Syndrome Characterized by Multiple Impairments: A Comprehensive Approach Is Needed. J Frailty Aging.

[8] Lee L, Patel T, Hillier LM, Maulkhan N, Slonim K, Costa A (2017). Identifying frailty in primary care: A systematic review. Geriatr Gerontol Int.

[9] Rockwood K, Mitnitski A (2007). Frailty in relation to the accumulation of deficits. J Gerontol A Biol Sci Med Sci.

[10] Blodgett J, Theou O, Kirkland S, Andreou P, Rockwood K (2015). Frailty in NHANES: Comparing the frailty index and phenotype. Arch Gerontol Geriatr.

[11] Hoogendijk EO, Rockwood K, Theou O, Armstrong JJ, Onwuteaka-Philipsen BD, Deeg DJH, Huisman M (2018). Tracking changes in frailty throughout later life: results from a 17-year longitudinal study in the Netherlands. Age Ageing.

[12] Tang Z, Wang C, Song X, Shi J, Mitnitski A, Fang X, Yu P, Rockwood K (2013). Co-occurrence of cardiometabolic diseases and frailty in older Chinese adults in the Beijing Longitudinal Study of Ageing. Age Ageing.

[13] Clegg A, Bates C, Young J, Ryan R, Nichols L, Ann Teale E, Mohammed MA, Parry J, Marshall T (2016). Development and validation of an electronic frailty index using routine primary care electronic health record data. Age Ageing.

[14] Boyd PJ, Nevard M, Ford JA, Khondoker M, Cross JL, Fox C (2019). The electronic frailty index as an indicator of community healthcare service utilisation in the older population. Age Ageing.

[15] Hsiao FY, Yang CL, Huang YT, Huang WF (2007). Using Taiwan's national health insurance research databases for pharmacoepidemiology research. J Food Drug Anal Jul.

[16] Searle SD, Mitnitski A, Gahbauer EA, Gill TM, Rockwood K (2008). A standard procedure for creating a frailty index. BMC Geriatr.

[17] Breiman L (2001). Random forests. Machine learning.

[18] Rockwood K, Song X, MacKnight C, Bergman H, Hogan DB, McDowell I, Mitnitski A (2005). A global clinical measure of fitness and frailty in elderly people. CMAJ.

[19] Rockwood K, Blodgett JM, Theou O, Sun MH, Feridooni HA, Mitnitski A, Rose RA, Godin J, Gregson E, Howlett SE (2017). A Frailty Index Based On Deficit Accumulation Quantifies Mortality Risk in Humans and in Mice. Sci Rep.

[20] Rockwood K, Mitnitski A (2011). Frailty defined by deficit accumulation and geriatric medicine defined by frailty. Clin Geriatr Med.

[21] Shin EK, Mahajan R, Akbilgic O, Shaban-Nejad A (2018). Sociomarkers and biomarkers: predictive modeling in identifying pediatric asthma patients at risk of hospital revisits. NPJ Digit Med.

[22] Mortazavi BJ, Bucholz EM, Desai NR, Huang C, Curtis JP, Masoudi FA, Shaw RE, Negahban SN, Krumholz HM (2019). Comparison of Machine Learning Methods With National Cardiovascular Data Registry Models for Prediction of Risk of Bleeding After Percutaneous Coronary Intervention. JAMA Netw Open.

[23] Zador Z, Landry A, Cusimano MD, Geifman N (2019). Multimorbidity states associated with higher mortality rates in organ dysfunction and sepsis: a data-driven analysis in critical care. Crit Care.

[24] Amaral R, Pereira AM, Jacinto T, Malinovschi A, Janson C, Alving K, Fonseca JA (2019). Comparison of hypothesis- and data-driven asthma phenotypes in NHANES 2007-2012: the importance of comprehensive data availability. Clin Transl Allergy.

[25] Previdelli AN, de Andrade SC, Fisberg RM, Marchioni DM (2016). Using Two Different Approaches to Assess Dietary Patterns: Hypothesis-Driven and Data-Driven Analysis. Nutrients.

