# Supplementary material for: Comparisons Between Hypothesis- and Data-Driven Approaches for Multimorbidity Frailty Index: A Machine Learning Approach
Source: J Med Internet Res. 2020 Jun 11;22(6):e16213. doi: 10.2196/16213 (PMC7317629; doi:10.2196/16213)
Supplement: Multimedia Appendix 1 [file jmir_v22i6e16213_app1.docx]

**Multimedia Appendix 1:** Model accuracy with various numbers of composite diseases

| Number of diseases  in model | Model accuracy | The difference of model accuracy between neighnors |
| --- | --- | --- |
| 25 | 0.5970432 | --- |
| 26 | 0.5975442 | 0.0005014797 |
| 27 | 0.5987123 | 0.0011676420 |
| 28 | 0.5987123 | 0.0000000000 |
| 29 | 0.5986299 | -0.0000823411 |
| 30 | 0.5987963 | 0.0001663340 |
| 31 | 0.5991290 | 0.0003326680 |
| 32 | 0.5987094 | -0.0004195515 |
| 33 | 0.6000454 | 0.0013360400 |
| 34 | 0.6004600 | 0.0004145962 |
| 35 | 0.6005440 | 0.0000839928 |
| 36 | 0.6005461 | 0.0000020647 |
| 37 | 0.6006268 | 0.0000806893 |
| **38** | **0.6022061** | 0.0015793470 |
| 39 | 0.6015437 | -0.0006624454 |
| 40 | 0.6010418 | -0.0005018926 |
| 41 | 0.6015441 | 0.0005023056 |
| 42 | 0.6015424 | -0.0000016517 |
| 43 | 0.6016277 | 0.0000852317 |
| 44 | 0.6012197 | -0.0004079891 |
| 45 | 0.6018817 | 0.0006620324 |
