# Supplementary material for: Comparisons Between Hypothesis- and Data-Driven Approaches for Multimorbidity Frailty Index: A Machine Learning Approach
Source: J Med Internet Res. 2020 Jun 11;22(6):e16213. doi: 10.2196/16213 (PMC7317629; doi:10.2196/16213)
Supplement: Multimedia Appendix 2 [file jmir_v22i6e16213_app2.docx]

**Multimedia Appendix 2:** Survival curves with 4 frail groups to depict calculation of distance index.


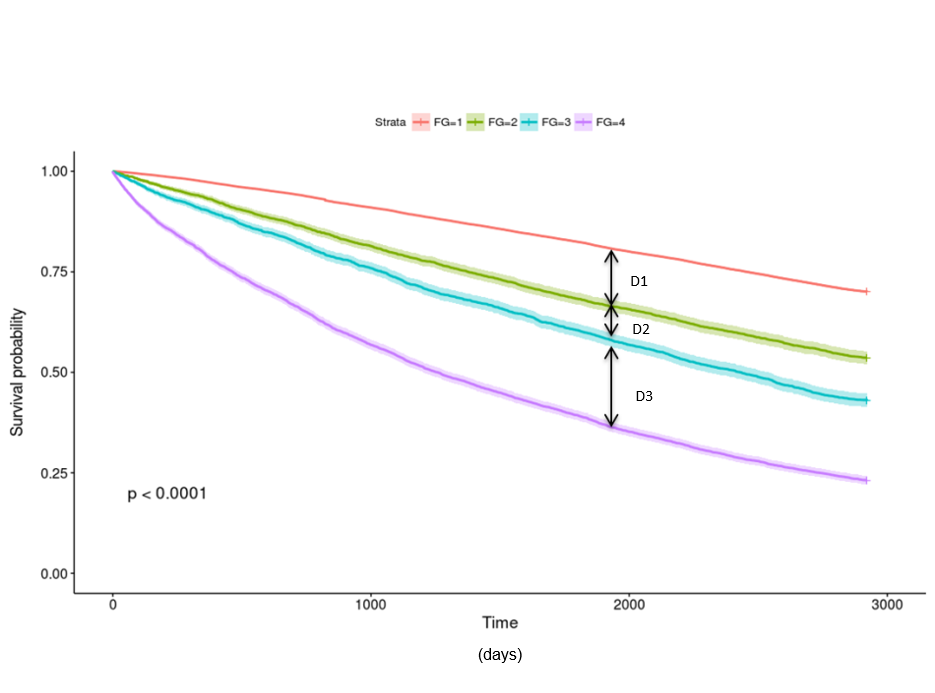


D1, D2 and D3 are distances with nearby group at a particular time point.
