# Supplementary material for: Comparisons Between Hypothesis- and Data-Driven Approaches for Multimorbidity Frailty Index: A Machine Learning Approach
Source: J Med Internet Res. 2020 Jun 11;22(6):e16213. doi: 10.2196/16213 (PMC7317629; doi:10.2196/16213)
Supplement: Multimedia Appendix 3 [file jmir_v22i6e16213_app3.docx]

**Multimedia Appendix 4:** Survival curves with 4 frail groups to depict calculation of coverage index.


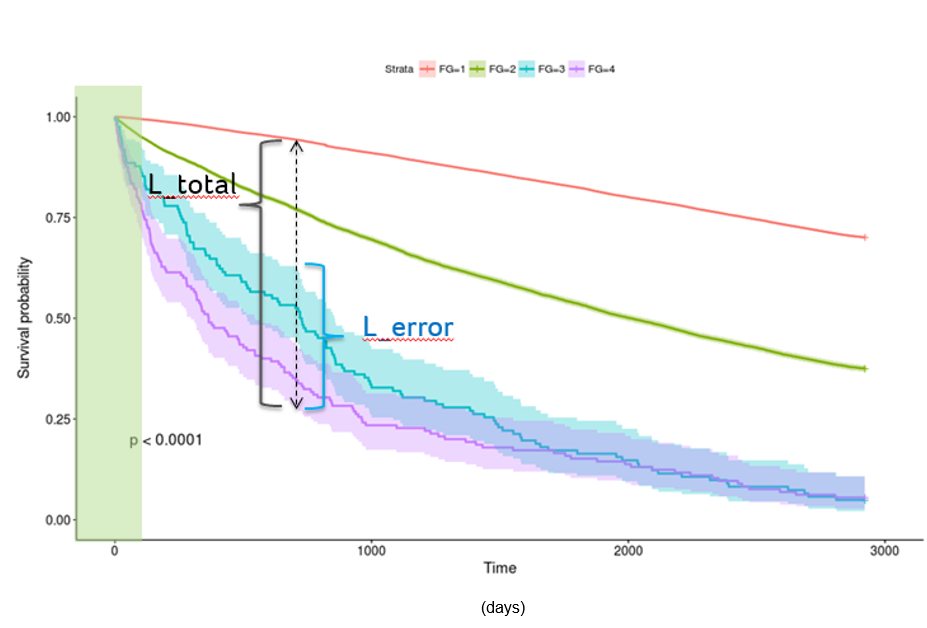


L_total measures the difference of estimated survival probability between fit group and server group. L_error measures the total estimated errors within 4 groups.
