# Supplementary material for: Comparisons Between Hypothesis- and Data-Driven Approaches for Multimorbidity Frailty Index: A Machine Learning Approach
Source: J Med Internet Res. 2020 Jun 11;22(6):e16213. doi: 10.2196/16213 (PMC7317629; doi:10.2196/16213)
Supplement: Multimedia Appendix 4 [file jmir_v22i6e16213_app4.docx]

**Multimedia Appendix 4:** Convergences and divergences between mFI and ML-mFI.

| In mFI, not in ML-mFI | In mFI, also in ML-mFI | In ML-mFI, not in mFI |
| --- | --- | --- |
| Entropion | Anemia | Diabetes mellitus |
| Meniere's disease | Senile dementia | Disorders of fluid electrolyte and acid-base balance |
| Cerebral atherosclerosis | Paralysis agitans | Other diseases of lung |
| Disorders of stomach | Hypertensive heart disease | Disorders of kidney and ureter |
| Gastroenteritis and colitis | Coronary artery disease | Fractures of intracapsular section of femur |
| Hypertrophy (benign) of prostate | Paroxysmal supraventricular tachycardia | Streptococcal septicemia |
| Contact dermatitis and other eczema | Congestive heart failure | Malignant neoplasm of liver |
| Pruritus | Cerebral thrombosis | Chronic liver diseases |
| Osteoporosis | Cognitive deficits, late effects of cerebrovascular disease | Intestinal obstruction |
| Renal colic | Acute, but ill-defined, cerebrovascular disease | Viral hepatitis |
|  | Simple chronic bronchitis | Secondary malignant neoplasm of kidney |
|  | Bronchial asthma | Streptococcus infections |
|  | Chronic airways obstruction | Gouty arthropathy |
|  | Diseases of esophagus | Hypertensive chronic kidney disease |
|  | Gastric ulcer | Malignant neoplasm of trachea, bronchus and lung |
|  | Duodenal ulcer | Secondary malignant neoplasm of lung |
|  | Constipation |  |
|  | Chronic renal failure |  |
|  | Urinary tract infection |  |
|  | Cellulitis and abscess, face |  |
|  | General symptoms |  |
|  | Tachycardia |  |
