# Supplementary material for: Comparisons Between Hypothesis- and Data-Driven Approaches for Multimorbidity Frailty Index: A Machine Learning Approach
Source: J Med Internet Res. 2020 Jun 11;22(6):e16213. doi: 10.2196/16213 (PMC7317629; doi:10.2196/16213)
Supplement: Multimedia Appendix 5 [file jmir_v22i6e16213_app5.docx]

**Multimedia Appendix 5**: Total number of deficits and the composing deficits of the ML-mFI in three subcohorts, including 65- to 75-year-olds, 76- to 85-year-olds and 85+-year-olds.

| **ML-mFI for age 65-75** | **ML-mFI for age 76-85** | **ML-mFI for age 86+** |
| --- | --- | --- |
| Total deficit number=**59** | Total deficit number=47 | Total deficit number=39 |
| Diabetes mellitus | Other disorders of urethra and urinary tract | Other disorders of urethra and urinary tract |
| Other disorders of urethra and urinary tract | Pneumonia_ organism unspecified | Pneumonia_ organism unspecified |
| Late effects of cerebrovascular disease | Other diseases of lung | Septicemia |
| Chronic liver disease and cirrhosis | Heart failure | Diabetes mellitus |
| Heart failure | Late effects of cerebrovascular disease | Disorders of fluid_ electrolyte and acid-base balance |
| Pneumonia_ organism unspecified | Diabetes mellitus | Essential hypertension |
| Chronic bronchitis | Disorders of fluid_ electrolyte and acid-base balance | Chronic bronchitis |
| Disorders of fluid_ electrolyte and acid-base balance | Chronic bronchitis | Other and unspecified anemias |
| Occlusion of cerebral arteries | Chronic airways obstruction_ not elsewhere classified | Chronic airways obstruction_ not elsewhere classified |
| Other disorders of kidney and ureter | Septicemia | Functional digestive disorders_ not elsewhere classified |
| Other diseases of lung | Cardiac dysrhythmias | Heart failure |
| Chronic airways obstruction_ not elsewhere classified | Essential hypertension | Other diseases of lung |
| Hypertensive heart disease | Other and unspecified anemias | Other disorders of kidney and ureter |
| Malignant neoplasm of liver and intrahepatic bile ducts | Osteoarthrosis and allied disorders | Other forms of chronic ischemic heart disease |
| Secondary malignant neoplasm of respiratory and digestive systems | Senile and presenile organic psychotic conditions | Gastric ulcer |
| Chronic renal failure | Gastrointestinal hemorrhage | Senile and presenile organic psychotic conditions |
| Secondary malignant neoplasm of other specified sites | Pleurisy | Pleurisy |
| Other forms of chronic ischemic heart disease | Other disorders of kidney and ureter | Chronic ulcer of skin |
| Viral hepatitis | Hyperplasia of prostate | Cardiac dysrhythmias |
| Fracture of neck of femur | Chronic renal failure | Cholelithiasis |
| Malignant neoplasm of trachea_ bronchus and lung | Chronic ulcer of skin | Other cellulitis and abscess |
| Other and unspecified anemias | Pneumonitis due to solids and liquids | Intestinal obstruction without mention of hernia |
| Septicemia | Other bacterial pneumonia | Duodenal ulcer |
| Essential hypertension | Symptoms involving cardiovascular system | Osteoarthrosis and allied disorders |
| Duodenal ulcer | Other forms of chronic ischemic heart disease | Other noninfectious gastroenteritis and colitis |
| Parkinson's disease | Acute renal failure | Pneumonitis due to solids and liquids |
| Hypertensive renal disease | Viral hepatitis | Fracture of neck of femur |
| Symptoms involving cardiovascular system | Hypertensive renal disease | Bronchopneumonia_ organism unspecified |
| Bacterial infection in conditions classified elsewhere and of unspecified site | Cataract | Late effects of cerebrovascular disease |
| Cardiac dysrhythmias | Chronic liver disease and cirrhosis | Parkinson's disease |
| General symptoms | Other and unspecified disorders of back | Nonspecific findings on examination of blood |
| Malignant neoplasm of rectum_ rectosigmoid junction_ and anus | Parkinson's disease | Gastrointestinal hemorrhage |
| Malignant neoplasm of bladder | Secondary malignant neoplasm of respiratory and digestive systems | Bacterial infection in conditions classified elsewhere and of unspecified site |
| Gastrointestinal hemorrhage | Malignant neoplasm of trachea_ bronchus and lung | Diseases of esophagus |
| Asthma | Disorders of lipoid metabolism | Conduction disorders |
| Gout | Other diseases of endocardium | Other bacterial pneumonia |
| Hyperplasia of prostate | Occlusion of cerebral arteries | Acute_ but ill-defined_ cerebrovascular disease |
| Other disorders of bone and cartilage | Gout | Diseases of mitral and aortic valves |
| Gastric ulcer | Bacterial infection in conditions classified elsewhere and of unspecified site | Gout |
| Intestinal obstruction without mention of hernia | Intestinal obstruction without mention of hernia |  |
| Diseases of esophagus | Other organic psychotic conditions (chronic) |  |
| Iron deficiency anemias | Disorders of plasma protein metabolism |  |
| Functional digestive disorders_ not elsewhere classified | Neurotic disorders |  |
| Secondary and unspecified malignant neoplasm of lymph nodes | Late effects of tuberculosis |  |
| Other symptoms involving abdomen and pelvis | Malignant neoplasm of liver and intrahepatic bile ducts |  |
| Purpura and other hemorrhagic conditions | Malignant neoplasm of rectum_ rectosigmoid junction_ and anus |  |
| Malignant neoplasm of stomach | Hypertensive heart disease |  |
| Other cerebral degenerations |  |  |
| Nonspecific findings on examination of blood |  |  |
| Intracerebral hemorrhage |  |  |
| Acute myocardial infarction |  |  |
| Pleurisy |  |  |
| Symptoms involving urinary system |  |  |
| Cataract |  |  |
| Arterial embolism and thrombosis |  |  |
| Late effects of tuberculosis |  |  |
| Other diseases of endocardium |  |  |
| Fracture of one or more tarsal and metatarsal bones |  |  |
| Senile and presenile organic psychotic conditions |  |  |
